# Cardiac autonomic function in REM-related obstructive sleep apnoea: insights from nocturnal heart rate variability profiles

**DOI:** 10.1007/s11325-024-03091-4

**Published:** 2024-07-01

**Authors:** Seren Ucak, Hasthi U. Dissanayake, Kate Sutherland, Brendon J. Yee, Kristina Kairaitis, John R. Wheatley, Amanda J. Piper, Philip de Chazal, Peter A. Cistulli, on behalf of the Sydney Sleep Biobank Investigators, Nina Sarkissian, Yu Sun Bin, Chin Moi Chow, Andrew Chan, Aimee Lowth, Jacob Graham, William Wood, Gary Cohen, Callum Bennett, Mohammad Ahmadi, Brendon Yee, Amanda Piper, Keith Wong, Kerri Melehan, Margaret Chan, David Wang, Gislaine Gauthier

**Affiliations:** 1 Charles Perkins Centre and Northern Clinical School, Faculty of Medicine and Health, University of Sydney, Sydney, Australia; 2 Department of Respiratory and Sleep Medicine, Royal North Shore Hospital, Sydney, Australia; 3 School of Biomedical Engineering, Faculty of Engineering, University of Sydney, Sydney, Australia; 4 Sydney Medical School, Faculty of Medicine and Health, University of Sydney, Sydney, Australia; 5 Woolcock Institute of Medical Research, Royal Prince Alfred Hospital, Sydney, Australia; 6 Ludwig Engel Centre for Respiratory Research, Westmead Institute for Medical Research, Sydney, Australia; 7 Department of Respiratory and Sleep Medicine, Westmead Hospital, Sydney, Australia; 8 Department of Respiratory and Sleep Medicine, Royal Prince Alfred Hospital, Sydney, Australia

**Keywords:** REM-related OSA, Obstructive sleep apnoea, Heart rate variability, Cardiac autonomic function

## Abstract

**Purpose:**

In light of the reported association between REM-related obstructive sleep apnoea (OSA) and heightened cardiovascular risk, this study aims to compare cardiac autonomic function in patients with REM-OSA and OSA independent of sleep stage. We hypothesized that REM-OSA patients would exhibit higher sympathetic cardiac modulation based on heart rate variability (HRV) profiles.

**Methods:**

HRV was compared between the OSA group (AHI ≥ 5 events/h, *n* = 252) and the REM-OSA group (AHI ≥ 5 events/h, AHIREM:AHINREM ≥ 2, *n* = 137). Time- and frequency-domain measures of HRV were analysed during N2 and REM sleep.

**Results:**

Clinical characteristics between the two test groups differed significantly, 45% of REM-OSA patients were female, with mild OSA (median, interquartile range (IQR)) AHI of 10 (7) events/h. Only 26% of the OSA cohort were female with moderate OSA (AHI = 17 (20) events/h, *p* < 0.001). Compared with the OSA group, the low frequency to high frequency ratio (LF:HF) and LF power were lower and HF power was higher in the REM-OSA group during N2 (LF:HF, *p* = 0.012; LF; *p* = 0.013; HF, p = 0.007) and in REM sleep (LF:HF, *p* = 0.002; LF, *p* = 0.004; HF, *p* < 0.001). Patient sex and OSA severity had a significant combined effect on average N to N interval, LF power, and LF:HF ratio during N2 and REM sleep (all *p* < 0.001).

**Conclusion:**

Contrary to our hypothesis, REM-OSA patients demonstrated consistently higher cardiac vagal modulation, reflecting better cardiac autonomic adaptation. These results were attributed to differences in OSA severity and sex in these two groups, both independently affecting HRV. This study emphasises the need for future research into the underlying pathophysiology of REM-OSA and the potential implications of sex and OSA severity on cardiovascular risk.

**Supplementary Information:**

The online version contains supplementary material available at 10.1007/s11325-024-03091-4.

## Introduction

Obstructive sleep apnoea (OSA), a highly prevalent and chronic sleep disorder, confers a heightened susceptibility to the development of hypertension, cardiovascular disease, and stroke. Repeated upper airway collapse results in intermittent hypoxia, negative intrathoracic pressure swings and sleep fragmentation [1, 2], causing adverse cardiovascular effects, primarily through sympathetic activation [3].

While respiratory events may occur in all stages of sleep, muscle hypotonia during rapid eye movement (REM) sleep is believed to play a role in the heightened frequency of upper airway collapse during that sleep stage [4]. This, in turn, leads to longer duration of respiratory events accompanied by greater oxygen desaturation and increased sympathetic activity [4, 5]. Therefore, OSA during REM sleep may have a significant effect on cardiovascular risk, even when the apnoea–hypopnoea index (AHI) is low [6].

There is evidence that OSA during REM sleep may be associated with cardiovascular changes that may confer increased cardiovascular risk. However, the mechanisms underpinning this association are not well characterised. Previous studies have focused on a single clinical outcome rather than investigating the physiological mechanisms underlying the increased cardiovascular risk associated with OSA during REM sleep. While some studies show an association between REM-related OSA (REM-OSA) and markers of cardiovascular risk, namely, hypertension and nocturnal blood pressure dipping [7], other studies show no association with clinical features [8]. Moreover, research suggests potential sex-based differences in cardiac autonomic function in patients with OSA [9], warranting further investigation into the role of sex in cardiovascular outcomes in this population.

The sympathetic and parasympathetic nervous system has a profound influence on heart rate and blood pressure control [4]. Heart rate variability (HRV) serves as a non-invasive assessment of beat–to–beat changes in cardiac autonomic control, providing an intermediatory marker of cardiovascular risk. HRV analysis is particularly valuable for assessing the balance between sympathetic and parasympathetic inputs to the cardiovascular system. A large body of evidence has shown that OSA is associated with a reduced HRV, indicative of cardiac sympathetic predominance [10], which is significantly linked to a two-fold rise in cardiovascular morbidity, acting as an independent cardiovascular risk factor [10, 11]. In this study we aimed to compare HRV profiles between REM-OSA and patients with upper airway obstruction independent of sleep stage. We hypothesised increased cardiovascular risk during sleep in REM-OSA compared to OSA patients, reflected by heightened cardiac sympathetic function and vagal withdrawal.

## Methods

### Participants

**Fig. 1:**
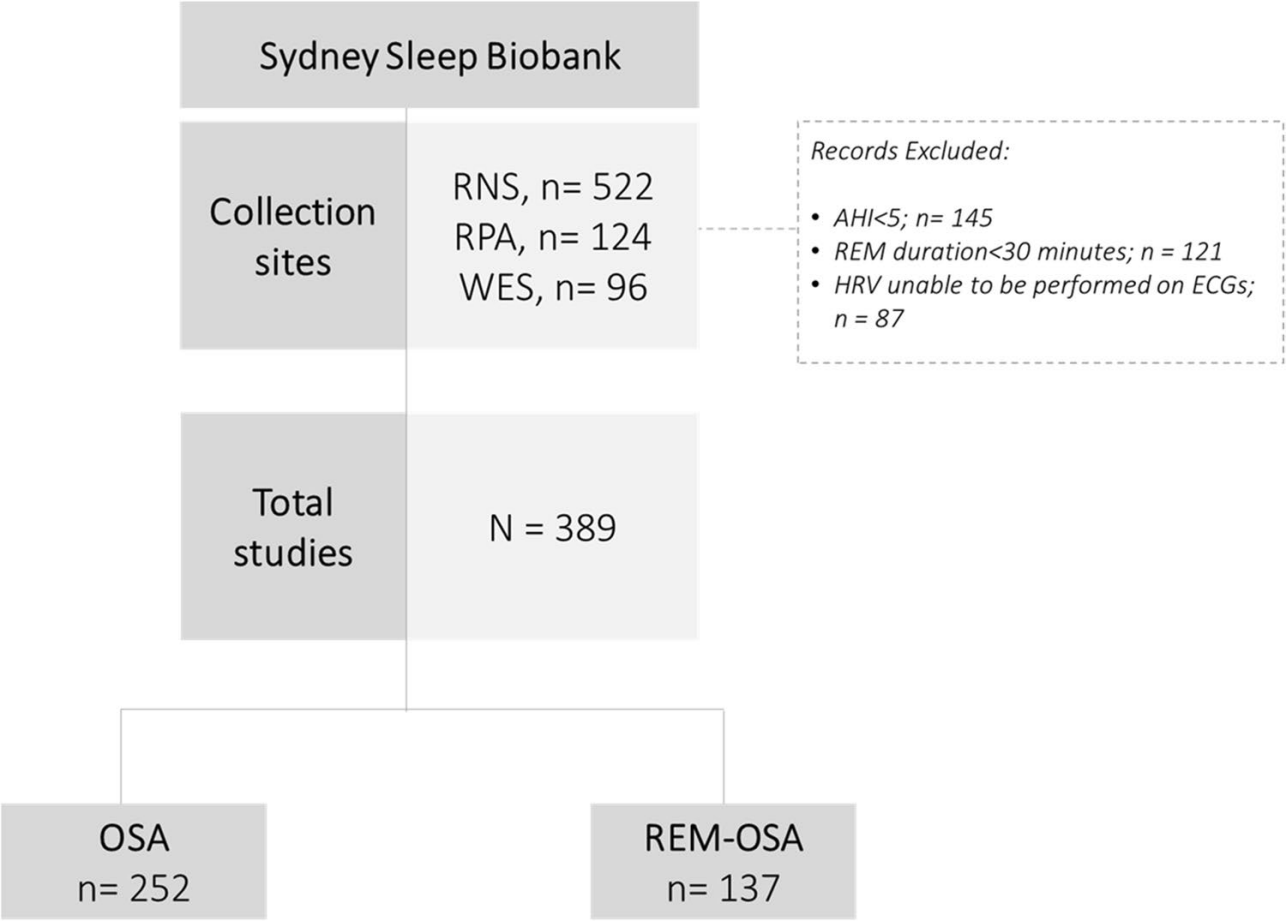
Study flow. Polysomnograms available from the Sydney Sleep Biobank were conducted at three collection sites located in Sydney, Australia: RNS (Royal North Shore Hospital), RPA (Royal Prince Alfred Hospital), and WES (Westmead Hospital). The REM-related OSA group (REM-OSA) included 137 patient records, while 252 patient records were included in the OSA independent of stage group (OSA)

We retrospectively reviewed polysomnograms (PSG) recorded in the Sydney Sleep Biobank, a database of prospective data collected from sleep clinic patients across 3 hospitals sleep clinics in Sydney, Australia (Fig. 1) [12]. As a part of their contribution to the Biobank, patients complete a subjective questionnaire responding to questions regarding ethnicity, comorbidities, and, medication use, and validated questionnaires including the Epworth Sleepiness Score (ESS), and Functional Outcomes of Sleep Questionnaire (FOSQ-10). The patients’ blood pressure was also taken pre- and post- polysomnography. Patients included in this study underwent nocturnal polysomnography at the Royal North Shore Hospital, Royal Prince Alfred Hospital and Westmead Hospital between 2018–2021. Patients required greater than 30 min of REM sleep to be included in the study. REM-related OSA (REM-OSA) can be sub-classified into two groups; (1) REM isolated OSA, defined as, AHI_REM_ > 5/ hour, AHI_NREM_ < 5/ hour, with Total AHI > 5/ hour; and (2) REM-predominant OSA, defined as AHI_REM_:AHI_NREM_ ≥ 2, with Total AHI > 5/ hour. While these definitions are distinct from one another they result in overlapping patient records, and therefore patient records pertaining to the latter, more broad definition of REM- predominant OSA was used as inclusion criteria for the REM-related OSA group (REM-OSA). The OSA group was defined as a Total AHI > 5 events/hour with exclusion of patients meeting our REM-OSA definition.

### Polysomnography

During full night PSG, standard channels were utilized to record electroencephalography (EEG), electrooculography (EOG), chin electromyography (EMG), nasal airflow pressure via nasal cannula, thoracic and abdominal respiratory effort, finger pulse oximetry to measure oxygen saturation (SpO_2_%), body position, and leg electromyography. Trained sleep scientists scored each recording according to the guidelines set by the American Academy of Sleep Medicine [1] and analyzed the data using the Compumedics PSG4 V4.1 software developed by Compumedics, Australia.

### Heart rate variability

The electrocardiogram was recorded as a part of in-clinic polysomnography and saved as a European data format (EDF). These recordings were imported into commercially available Sentinel software (Spacelabs Healthcare, Issaquah, WA, U.S.A.). The Sentinel software then processed the electrocardiogram by applying an automated R wave and electrocardiogram artefact detection algorithms and then exported an extensible markup language (xml) file containing the QRS times of heart-beats (with a 1ms resolution) and associated heart-beat labels (normal or non-normal beat/rhythm). Sentinel beat analysis files were imported into MATLAB software as xml files.

As part of in-laboratory PSG, electrocardiogram data was collected using three electrodes placed in the standard lead II configuration. The ECG signals were extracted from the polysomnograms and analysed using commercially available Holter software (Sentinel Holter Data Management System v11.5.1, Spacelabs Healthcare, Issaquah, WA, U.S.A.) for QRS detection, ectopic beat detection, and labeling. QRS detection was performed with a resolution of 1ms. An in-house algorithm was used to form the RR series [13]. The normal-normal (NN) beat series was derived from the RR series by using an accepted adaptive RR interval filtering algorithm method to remove RR intervals associated with non-sinus beats [14]. Time domain measures were calculated according to standard guidelines [15]. To calculate frequency-domain HRV parameters the Lomb periodogram was applied to the preprocessed data. The Lomb periodogram is based upon the same fundamental theory as the Discrete Fourier Transform but is superior as it does not require an evenly sampled data set – it allows for the inherent variability of the RR interval data and hence the tachogram can be transformed directly without an intervening approximation stage [16]. Unlike the Discrete Fourier Transform, the Lomb method also allows for the exclusion of ectopic beats. Time and frequency domain HRV measures used in this study are outlined in Table 1. HRV parameters were calculated using 2 min blocks, shifted by 30 s, across the entire ECG signal, and then averaged across sleep stage N2 and REM (Supplementary material Fig. 1 details the number of blocks of data analysed for each sleep stage).

**Table 1:** Descriptions and physiological interpretations of heart rate variability (HRV)

	Description	Physiological interpretation
Heart Rate Variability		
Average NN Interval, ms	Average time between consecutive R-peaks	Primarily parasympathetic cardiac modulation
SDNN, ms	Standard deviation of normal to normal intervals	Global HRV measure
RMSSD, ms	Root mean square of successive RR interval differences	Primarily parasympathetic cardiac modulation
pNN50, %	Percentage of successive RR interval that differ by more than 50 ms	Primarily parasympathetic cardiac modulation
TP, ms^2^	The absolute power of the frequency spectrum, excluding the very low frequency band (0.04–0.4Hz)	Global HRV measure
VLF, ms^2^	Absolute power of the very low frequency band (< 0.04Hz)	Link with thermoregulation and cardiac humoral influence
LF, ms^2^	Absolute power of the low-frequency band (0.04–0.15 Hz)	Sympathetic with a parasympathetic component
HF, ms^2^	Absolute power of the high-frequency band (0.15–0.40 Hz)	Primarily parasympathetic cardiac modulation
LF:HF	Ratio of LF-to-HF power	Sympathovagal balance
LF, nu	Relative power of the low-frequency band (0.04–0.15 Hz) in normal units	Primarily sympathetic cardiac modulation
HF, nu	Relative power of the high-frequency band (0.15–0.4 Hz) in normal units	Primarily parasympathetic cardiac modulation

### Statistical analysis

Clinical data were summarised across the test groups. The Shapiro–Wilk test was used to assess normality. All outcomes were non-parametric and presented as median (interquartile ranges, IQR). Categorical variables were expressed as count (percentage, %) and examined using Chi Square tests, presented with Chi square test statistic, χ^2^, and degrees of freedom (df). The Mann–Whitney U test compared clinical, polysomnographic, and HRV measures between the OSA and REM-OSA groups.

Linear regression models were employed to investigate the effect of OSA severity, patient sex, and test groups on HRV measures during N2 and REM sleep. Each HRV measure was considered a dependent variable, and the predictors for each model are as follows: Model 1 included AHI; Model 2 included sex and AHI; and Model 3 also incorporated grouping, i.e., OSA or REM-related OSA. Since continuous variables exhibited non-normal distributions, HRV markers were logarithmically transformed using Log10 (HRV + 1). + 1 was included in the logarithm parameters to permit analysis of zero values [17].

The p-value was adjusted for multiple comparisons (which included average NN interval, RMSSD, pNN50, TP, absolute LF and HF power, normalised LF and HF power, and LF:HF ratio) using Bonferroni correction. Therefore, significance is indicated by p-values less than 0.005. All data analyses were conducted using SPSS software (version 24; SPSS Inc., Chicago, IL).

## Results

### Comparison of phenotypic characteristics between OSA groups

There were no significant differences in age, body mass index (BMI), blood pressure, ESS, or FOSQ-10 scores observed across the three study groups (Table 2). Females comprised approximately half of the REM-OSA group (45%), a proportion significantly higher than the OSA group, which consisted of 26% females (*p* < 0.001). Roughly half of the participants in each group self-identified as White (*p *= 0.031). In the REM-OSA group, there was a trend towards a higher proportion of participants identifying as North/North East Asian (*p* = 0.057). Subjective reports of hypertension were comparable across groups, with around one-third of participants reporting this condition (OSA, 41%; REM-OSA 29%; *p* = 0.042), as well as hypercholesterolaemia (OSA, 38%; REM-OSA 31%; *p* = 0.245). Notably, a larger proportion of participants in the OSA group reported using β-blockers (*p* = 0.013) and lipid-modifying agents (*p* = 0.029).

**Table 2:** Clinical Characteristics of OSA (*n* = 252) and REM-OSA (*n* = 137) Groups

Clinical Characteristics	OSA	REM-OSA		
	*N* = 252	*N* = 137	*Test statistic*	*p*
Age, years	56 (21)	55 (25)	-0.29	0.772
BMI, kg/m^2^	29 (8)	29 (7)	-1.04	0.295
Sex, female count (%)	66 (26)	62 (45)	14.61 (1)	< 0.001*
Night-time Systolic BP, mmHg	126 (24)	126 (24)	-1.12	0.261
Night-time Diastolic BP, mmHg	78 (12)	79 (12)	-0.31	0.753
Daytime Systolic BP, mmHg	123 (24)	124 (23)	-0.75	0.453
Daytime Diastolic BP, mmHg	77 (14)	77 (13)	-1.41	0.158
ESS	7 (7)	6 (8)	-1.17	0.242
FOSQ10	16 (4)	17 (5)	-1.03	0.305
Ethnicity				
Caucasian, count (%)	166 (66)	75 (55)	4.66 (1)	0.031
Indigenous Australian, count (%)	6 (2)	3 (2)	0.01 (1)	0.905
South/ South East Asian, count (%)	33 (13)	14 (10)	0.02 (1)	0.904
North/ North East Asian, count (%)	10 (4)	13 (10)	3.6 (1)	0.057
Middle Eastern/ North African, count (%)	5 (2)	7 (5)	2.9 (1)	0.089
Comorbidities				
Diabetes, count (%)	41 (16)	15 (11)	2.42 (1)	0.289
Hypertension, count (%)	104 (41)	40 (29)	6.35 (1)	0.042
Hypercholesterolaemia, count (%)	96 (38)	43 (31)	2.81 (1)	0.245
Coronary artery disease, count (%)	25 (10)	8 (5)	3.64 (1)	0.161
Depression, count (%)	64 (25)	26 (19)	2.33 (1)	0.312
Anxiety, count (%)	61 (24)	23 (17)	4.34 (1)	0.114
Medication use				
β-blocker, count (%)	11 (4)	0 (0)	6.15 (1)	0.013
ACEI or ARB, count (%)	36 (14)	12 (9)	2.51 (1)	0.113
Lipid modifying agents, count (%)	30 (12)	7 (5)	4.76 (1)	0.029
Antidepressant or anti-anxiety, count (%)	22 (9)	12 (9)	0.00 (1)	0.992

All continuous variables were analysed nonparametrically using the Wilcoxon-Mann–Whitney test. Data are displayed as median and interquartile range (IQR) along with the test statistic (U). Categorical variables were compared using Chi-Square tests, and results are presented as count (percentage, %) along with the Chi-Square test statistic and degrees of freedom (χ2(df)). *P* values underwent adjustment for multiple comparisons using Bonferroni correction. Significance is indicated *as p < 0.005

*BMI* body mass index, *BP* blood pressure, *ESS* Epworth sleepiness scale, *FOSQ10* Functional outcomes of sleep questionnaire-10, *ACEI* Angiotensin-converting enzyme inhibitors, *ARB* Angiotensin II receptor blockers

### Comparison of polysomnographic characteristics

The REM sleep stage was longer in the REM-OSA group (*p* = 0.008) (Table 3). By design, the AHI varied significantly between test groups, where the REM-OSA group had an AHI of 10 (7) events/h, compared with 17 (20) events/h in the OSA group (*p* < 0.001). The AHI_NREM_ was significantly higher in the OSA group (*p* < 0.001), whereas the AHI_REM_ was significantly higher in the REM-OSA group (*p* < 0.001). There were no differences in SpO_2_ nadir; however, SpO_2_Nadir NREM was highest in the REM-OSA group (*p* < 0.001).

**Table 3:** Polysomnographic characteristics of OSA (*n* = 252) and REM-OSA (*n* = 137) Groups

	OSA	REM-OSA		
Polysomnographic characteristics	*N* = 252	*N* = 137	*Test statistic*	*P*
Sleep latency, min	21 (26)	17 (25)	-1.32	0.184
REM latency, min	118 (101)	96 (70)	-2.67	0.007
Sleep efficiency, %	78 (17)	78 (17)	-1.19	0.233
Total Sleep Time, min	356 (100)	358 (77)	-1.25	0.209
N2, min	183 (70)	196 (71)	-2.02	0.043
REM, min	55 (36)	65 (37)	-2.66	0.008
AHI, events/hr	17 (20)	10 (7)	-8.54	< 0.001*
AHI_NREM_, events/hr	22 (27)	7 (8)	-12.07	< 0.001*
AHI_REM_, events/hr	20 (26)	36 (21)	-5.05	< 0.001*
AHI_REM:NREM_	0.8 (0.9)	4.2 (5.6)	-16.28	< 0.001*
ODI 3%, events/hr	15 (21)	9 (8)	-6.14	< 0.001*
SpO_2_ nadir, %	86 (9)	84 (7)	-0.74	0.458
SpO_2_ nadir NREM, %	87 (7)	88 (4)	-4.67	< 0.001*
SpO_2_ nadir REM, %	88 (9)	85 (8)	-0.46	0.639
SpO_2_ < 90%	0.5 (4.4)	0.7 (1.9)	-1.55	0.121

All continuous variables were nonparametric. Differences between groups were assessed using the Wilcoxon-Mann–Whitney test. Data are presented as median and interquartile range (IQR) with the test statistic. P values were adjusted for multiple comparisons using Bonferroni correction. Significance is denoted *as *p* < 0.005

*REM*- rapid eye movement, *N2*- non rapid eye movement stage 2 sleep, *AHI* apnoea hyponoea index, *ODI* oxygen desaturation index, *SpO*_*2*_ peripheral oxygen saturation

### Cardiac autonomic activity across sleep stages

**Fig. 2:**
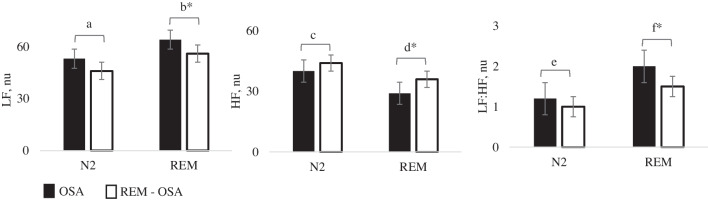
Bar Graphs Illustrating Selected HRV Measures in OSA (*n* = 235, black bars) and REM-OSA Patients (*n* = 137, white bars). Wilcoxon-Mann–Whitney test was employed for statistical analysis. Data are presented as median and interquartile range (IQR) along with the test statistic (U). P values were adjusted for multiple comparisons using Bonferroni correction. Significance is indicated by for *p* < 0.005. a, *p* = 0.012; b, *p* = 0.002; c, *p* = 0.013; d, *p* = 0.004; e, *p* = 0.007; f, *p* < 0.001. Abbreviations: LF—low-frequency power; HF—high-frequency power; LF: HF—low-frequency: high-frequency ratio.; nu—normalised units

Compared with the OSA group, LF power (*p* = 0.013), and the LF:HF ratio (*p* = 0.012) was lower while HF power (*p* = 0.007) was higher in REM-OSA during N2 sleep (Table 4). This relationship continued into REM sleep and was significantly different between both groups (LF, *p* = 0.002; LF:HF ratio, *p* = 0.004; HF, *p* < 0.001) (Fig. 2).

**Table 4:** Heart rate variability measures during N2 and REM sleep of OSA (*n* = 252) and REM-OSA (*n* = 137) Groups

HRV	OSA	REM-OSA		
	*N* = 252	*N* = 137	*Test Statistic*	*P*
N2				
avgNN _ms_	992 (244)	990 (224)	-0.75	0.451
SDNN _ms_	42 (31)	42 (24)	-0.59	0.554
RMSSD _ms_	33 (28)	34 (35)	-0.47	0.635
pNN50^%^	10 (22)	11 (25)	-0.42	0.671
TP _ms_^2^	1957 (3020)	1878 (2970)	-0.38	0.701
LF _ms_^2^	536 (1152)	447 (922)	-0.93	0.352
HF _ms_^2^	428 (836)	470 (1037)	-0.92	0.353
LF: HF	1.2 (2.1)	1.0 (0.1)	-2.52	0.012
LF_nu_	53 (31)	46 (24)	-2.48	0.013
HF_nu_	40 (26)	44 (24)	-2.70	0.007
REM				
avgNN _ms_	967 (215)	956 (178)	-0.38	0.699
SDNN _ms_	44 (38)	45 (35)	-0.02	0.982
RMSSD _ms_	24 (23)	27 (32)	-0.99	0.322
pNN50^%^	4 (16)	6 (20)	-1.37	0.168
TP _ms_^2^	2193 (4281)	2305 (3551)	-0.18	0.857
LF _ms_^2^	492 (1026)	453 (1014)	-0.53	0.596
HF _ms_^2^	198 (553)	300 (698)	-1.15	0.248
LF: HF	2.0(2.8)	1.5 (1.7)	-3.15	0.002*
LF_nu_	64 (28)	56 (26)	-2.89	0.004*
HF_nu_	29 (22)	36 (20)	-3.50	< 0.001*

All continuous variables were nonparametric. Differences between groups were assessed using the Wilcoxon-Mann–Whitney test. Data are presented as median and interquartile range (IQR) with the test statistic. P values were adjusted for multiple comparisons using Bonferroni correction. Significance is denoted *as *p* < 0.005

*Avg NN *average NN interval, *SDNN *standard deviation of NN intervals, *RMSSD *root mean square of successive differences, *pNN50% *percentage of successive NN intervals differing by more than 50 ms, *TP *total power, *LF *low-frequency power, *HF *high-frequency power, *LF*: *HF *low-frequency: high-frequency ratio

### The effect of covariates on HRV

Linear regression models included OSA severity, sex, and test group as predictors. While no significant linear relationship was observed with AHI and HRV markers, the model explained 3% of the variance in normalised HF power during REM sleep (*p* = 0.003) (Table 5). When patient sex was introduced, significant linear relationships were identified. The combined model accounted for approximately 5–6% of the variance in absolute LF, LF:HF ratio, and normalised LF (all *p* < 0.001), as well as HF power during both N2 (*p* = 0.002) and REM sleep (*p* = 0.007). The combination of AHI, sex, and test group did not appear to have an effect on HRV measures, except for its contribution to 7% of the variance in LF:HF ratio during REM sleep (*p* < 0.001).

**Table 5:** Linear Regression Models Assessing Predictors (AHI, Gender, and Test Group) on HRV in OSA (*n* = 252) and REM-OSA (*n* = 137) Patients during N2 and REM Sleep

HRV	Model 1			Model 2			Model 3		
	*β (SE)*	*R*^*2*^	*P*	*β (SE)*	*R*^*2*^	*p*	*β (SE)*	*R*^*2*^	*P*
N2									
avgNN _ms_	0.00 (0.00)	0.08	< 0.001*	0.00 (0.00)	0.13	< 0.001*	-0.00 (0.00)	0.13	0.287
SDNN _ms_	0.00 (0.00)	0.00	0.700	0.00 (0.00)	0.01	0.046	0.00 (0.02)	0.01	0.799
RMSSD _ms_	0.00 (0.00)	0.00	0.313	0.00 (0.00)	0.01	0.307	0.01 (0.02)	0.01	0.486
pNN50^%^	0.00 (0.00)	0.00	0.320	-0.06 (0.06)	0.01	0.310	0.01 (0.06)	0.01	0.845
TP _ms_^2^	0.00 (0.00)	0.00	0.855	-0.10 (0.05)	0.01	0.061	-0.01 (0.06)	0.01	0.914
LF _ms_^2^	0.00 (0.00)	0.00	0.629	-0.20 (0.06)	0.03	< 0.001*	-0.03 (0.06)	0.03	0.680
HF _ms_^2^	0.00 (0.00)	0.01	0.128	-0.04 (0.07)	0.01	0.542	0.05 (0.07)	0.01	0.516
LF: HF	0.00 (0.00)	0.02	0.011	-0.08 (0.02)	0.05	< 0.001*	-0.04 (0.02)	0.06	0.063
LF_nu_	0.00 (0.00)	0.00	< 0.001*	-0.09 (0.02)	0.05	< 0.001*	-0.00 (0.02)	0.06	0.160
HF_nu_	0.00 (0.00)	0.03	0.001	0.07 (0.02)	0.05	0.002*	0.04 (0.02)	0.06	0.072
REM									
avgNN _ms_	0.00 (0.00)	0.04	< 0.001*	-0.02(0.01)	0.10	< 0.001*	0.00 (0.04)	0.10	0.953
SDNN _ms_	0.00 (0.00)	0.00	0.629	-0.01(0.02)	0.02	0.027	0.04 (0.00)	0.02	0.116
RMSSD _ms_	0.00 (0.00)	0.00	0.511	0.00(0.00)	0.01	0.480	0.00 (0.00)	0.01	0.084
pNN50^%^	0.00 (0.01)	0.00	0.921	-0.11 (0.07)	0.01	0.087	0.13 (0.07)	0.02	0.068
TP _ms_^2^	0.01 (0.02)	0.00	0.529	-0.19 (0.06)	0.03	0.002*	0.07 (0.07)	0.04	0.299
LF _ms_^2^	0.0 (0.02)	0.00	0.942	-0.30 (0.07)	0.06	< 0.001*	0.05 (0.07)	0.06	0.538
HF _ms_^2^	-0.02 (0.03)	0.00	0.469	-0.12 (0.08)	0.01	0.140	0.14 (0.09)	0.02	0.104
LF: HF	0.02 (0.01)	0.02	0.018	-0.10 (0.03)	0.06	< 0.001*	-0.10 (0.03)	0.07	< 0.001*
LF_nu_	0.00 (0.01)	0.00	0.555	-0.09 (0.02)	0.06	< 0.001*	-0.03 (0.02)	0.07	0.235
HF_nu_	-0.02 (0.01)	0.03	0.003*	0.08 (0.02)	0.06	0.007	0.06 (0.03)	0.08	0.015

All continuous variables were non-parametric and as such were log10 + 1 transformed prior to linear regression and are presented as Median (IQR). The unstandardised beta (standard error) and R^2^ statistic are shown for each calculation. P-values were adjusted for multiple comparisons using Bonferroni correction; significance is indicated by as p < 0.005

Dependent variables for each model are HRV measures. Predictors in: Model 1 include AHI; Model 2 includes gender and AHI; Model 3 include AHI, gender, and test group (OSA or REM-OSA)

*Avg NN *average NN interval, *SDNN *standard deviation of NN intervals, *RMSSD *root mean square of successive differences, *pNN50% *percentage of successive NN intervals differing by more than 50 ms, *TP *total power, *LF *low-frequency power, *HF *high-frequency power, *LF: HF *low-frequency: high-frequency ratio

## Discussion

This study explored nocturnal cardiac autonomic profiles of patients with REM-OSA to a group with OSA independent of sleep stage. The primary finding in this study is that compared with REM-OSA, OSA patients’ have a predominance of sympathetic cardiac modulation during both N2 and REM sleep stages. There was a significantly higher percentage of females with REM-OSA (45%) when compared with the OSA group (26%). We also demonstrated that while OSA severity has a limited effect of HRV measures, the combination of AHI and patient sex accounts for up to 6% in variance of sympathetically mediated HRV markers in both sleep stages.

We hypothesised increased sympathetic and reduced parasympathetic cardiac autonomic function in patients with REM-OSA, as a marker of increased cardiovascular risk. Our results, while not supporting our hypothesis, are consistent with available literature [18]. Of the 3 similar studies undertaken, only one study has reported that apnoea during REM sleep is associated with sympathetic predominance. The authors link higher OSA severity during REM sleep to increased low frequency power and LF:HF ratio in wakefulness [17]. Earlier work shows a dose–response relationship between OSA severity and absolute LF power in men and women during REM sleep and wakefulness. Interestingly, BMI inversely affects low frequency and total power during REM sleep (and no other sleep stage) [19]. Only one study provides an assessment of HRV in patients with REM-OSA [18], and found no evidence of increased cardiovascular risk in patients with REM-OSA compared with matched control and OSA groups. In fact, their results show significantly greater SDNN, a marker for global HRV and therefore lower cardiovascular risk [15], in REM-OSA patients when compared with an OSA group. Our results show that compared with OSA patients, REM-OSA have higher absolute HF power and lower LF:HF ratio during sleep. This is associated with greater cardiac vagal modulation and ultimately reduced cardiovascular risk [15]. Not to mention, the greater use of ACEI, ARB and lipid modifying agents in the OSA group, which also effect HRV [20]. Taken together, these studies provide evidence of distinct cardiac autonomic function profiles in patients with REM-OSA, though there remains disagreement on the direction of sympathovagal balance and therefore its impact on cardiovascular risk.

Altered cardiac autonomic control in sleep is affected by the degree of OSA severity. When shifting from NREM to REM sleep, HRV profiles of patients with moderate OSA are characterised by significantly greater cardiac sympathetic activity (increased LF and LF:HF components) coupled with reduced cardiac vagal activity (reduced HF spectral components) [21]. The genesis of this being related to sympathoexcitation which peaks towards the end of the respiratory event and is followed by a surge in blood pressure and heart rate repeatedly experienced by OSA patients throughout the duration of sleep. This ultimately contributes to the ‘non-dipping’ nocturnal blood pressure profiles commonly exhibited in OSA resulting in susceptibility to hypertension. In fact increased bursts of muscle sympathetic nerve activity have been reported during REM sleep of patients with OSA potentially contributing to cardiovascular sequalae [4]. In both of our OSA groups the SDNN falls below the clinically relevant threshold of > 50ms, which is strongly associated with cardiovascular mortality in patients with pre-existing cardiovascular disease [22].

The observed reduction in cardiac sympathetic activity among our REM-OSA group can, in part, be attributed to variations in OSA severity between the test groups. Notably, our REM-OSA cohort had a higher proportion of patients with mild OSA, which aligns with findings in existing literature. Studies have shown that about 70% of sleep studies with an overall AHI < 15 events/h (indicating either no OSA or mild OSA) are affected by REM-OSA, defined as REM AHI ≥ 15 events/h [23]. While no linear relationships were identified between AHI and HRV markers, our modelling did reveal that OSA severity explained approximately 3% of the variance in HF power during REM sleep. This likely contributed to a shift in the sympathovagal balance towards vagal predominance in the REM-OSA group.

The impact of sex on cardiovascular risk in patients with OSA also requires attention due to its potential implications for understanding the underlying pathophysiological consequences of OSA. Generally speaking, there are fewer HRV studies conducted in women than there are in men. Kesek and colleagues studied the relationship between OSA severity and HRV in 387 women and showed an inverse relationship between AHI and sympathetic HRV markers during REM sleep [9]. More recent work has confirmed a marked difference in OSA prevalence between women pre and post menopause, suggesting that comparing patients simply by sex does not accurately characterise an OSA phenotype [24]. The role that reproductive hormones play in OSA remains an underexplored area of study. Lower levels of progesterone, oestradiol, and 17-OH progesterone is noted in post-menopausal women with an AHI > 10 events/h, after matching for age, and menstrual time-point [25]. In fact, hormone replacement therapy in post-menopausal women appears to lower AHI significantly when compared with women not on therapy, though the absolute difference in AHI remains low (∼ 2 events/h) [24]. Our study showed that the combination of patient sex and OSA severity contributed to heightened cardiac vagal activity in REM-OSA patients during N2 sleep. Furthermore, when we factored in patient sex, we found that it explained a greater portion (up to 6%) of variance in global and sympathetically mediated HRV measures during REM sleep. This aligns with findings in related studies [9, 18], suggesting that the diminished cardiovascular risk observed in REM-OSA may be attributed to a dual effect: lower overall AHI severity and a higher proportion of female patients. Given the older average age of the test group it would have been beneficial to study the impact of reproductive hormones on the presence and severity of REM-related OSA. This is a major limitation to this study, which we believe will be important to consider in future investigations.

Nevertheless, our study found that one third of all OSA patients, irrespective of test group, were diagnosed with hypertension (OSA, 41%; REM-OSA, 29%) and hypercholesterolaemia (OSA, 38%; REM-OSA, 31%). This is in line with the literature, where severe REM-OSA is independently associated with the prevalence of hypertension [6], incidence of a composite cardiovascular endpoints [26], and impairment of glucose metabolism [23, 27]. Inconsistency within the literature is also likely related to the emphasis on clinical outcomes rather than understanding the underlying physiological differences in REM-OSA.

The major strength of our study is the quantitative analysis of the effect of autonomic dysfunction, a potential mechanism underpinning cardiovascular risk in patients with OSA. We controlled for patient sex and OSA severity, statistically different between test groups, which enabled us to highlight the importance in considering these factors in this clinical subtype. We used a conservative HRV methodology which excluded epochs with mixed sleep stages and ECG sections associated with respiratory events or cardiac arrhythmia and entire epochs were excluded if the total exclusion period exceeded 12 s (10% of epoch length). We also included only patients with a minimum REM duration of 30 min to ensure that the count of studied epochs was comparable across test groups. The study has a number of limitations. Firstly, the imbalanced sample sizes between the test groups potentially limited the statistical power to detect significant differences or associations between variables. Secondly, patients were only studied during sleep due to a lack of wakefulness data. Comparison with daytime wakeful HRV measures would have allowed us to deduce the “spillover” effect of different nocturnal HRV profiles into daytime cardiac autonomic function.

## Conclusion

This comparative analysis of nocturnal HRV in REM-OSA and OSA independent of sleep stage found that REM-OSA patients consistently showed increased cardiac vagal modulation, indicating enhanced autonomic adaptability. Notably, differences in sex and OSA severity between groups independently influenced HRV profiles. Recognising and accounting for these factors is imperative when evaluating HRV in OSA patients. This study emphasises that, beyond OSA severity, sex can also play a significant role in influencing cardiac autonomic adaptability.

## Supplementary Information

Below is the link to the electronic supplementary material.

Supplementary file1 (DOCX 85 KB)

## Data Availability

Access to the datasets for this study may be available if permissible by the Sydney Sleep Biobank. To view generated analyses and request access to datasets please confer with corresponding author.
