# Supplementary material for: Cardiac autonomic function in REM-related obstructive sleep apnoea: insights from nocturnal heart rate variability profiles
Source: Sleep Breath. 2024 Jul 1;28(5):1987–96. doi: 10.1007/s11325-024-03091-4 (PMC11450088; doi:10.1007/s11325-024-03091-4)
Supplement: Supplementary file 1 — Supplementary file1 (DOCX 85 KB) [file 11325_2024_3091_MOESM1_ESM.docx]

# SUPPLEMENTARY MATERIAL


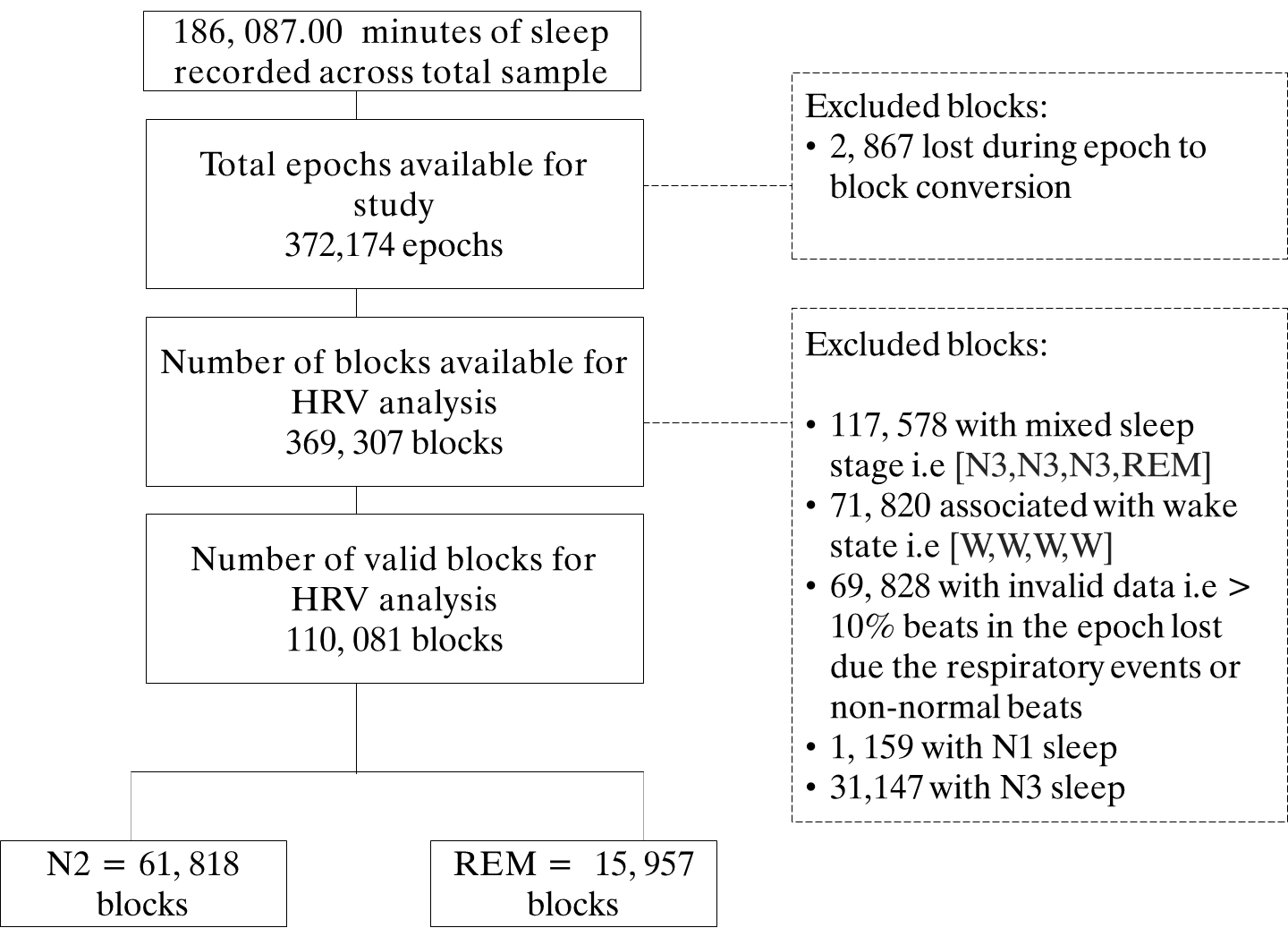
**Figure 1: Flowchart of epochs used in HRV analysis**

Of the 389 patient ECG records a total of 186, 087 minutes’ worth of sleep data was available for analysis, this equates to 372, 174 (30 second) epochs. A number of epochs were excluded due to mixed sleep stage, epoch – to – block conversion and invalid data e.g. segments with respiratory events or ectopic beats. Epochs are considered 30 second segments of ECG data taken from the PSG. Blocks are considered 30 second R to R segments which make up larger 2-minute segments, shifting by 30 seconds for HRV analysis.

Abbreviations: HRV – heart rate variability, ECG – electrocardiography, PSG – polysomnogram, REM – rapid eye movement, N1-N3 – Non-REM sleep stage 1-3, W - wakefulness
